# Association between Adverse Reactions and Humoral Immune Response No Longer Detectable after BNT162b2 Booster Vaccination

**DOI:** 10.3390/vaccines10101608

**Published:** 2022-09-25

**Authors:** Stilla Bauernfeind, Sebastian Einhauser, Leonid Tydykov, Anna-Lena Mader, Bernd Salzberger, Florian Hitzenbichler, Arno Mohr, Ralph Burkhardt, Ralf Wagner, David Peterhoff

**Affiliations:** 1 Department of Infection Prevention and Infectious Diseases, University Medical Center Regensburg, 93053 Regensburg, Germany; 2 Institute for Medical Microbiology and Hygiene, University of Regensburg, 93053 Regensburg, Germany; 3 Center for Pneumology, Donaustauf Hospital, 93093 Donaustauf, Germany; 4 Institute of Clinical Chemistry and Laboratory Medicine, University Medical Center Regensburg, 93053 Regensburg, Germany; 5 Institute for Clinical Microbiology and Hygiene, University Medical Center Regensburg, 93053 Regensburg, Germany

**Keywords:** COVID-19, vaccination, BNT162b2, reactogenicity, immunogenicity, booster

## Abstract

In a previous study, we described a highly significant association between reactogenicity and SARS-CoV-2 RBD IgG titers and wild-type neutralization capacity in males after basic vaccination with BNT162b2. The objective of this study was to assess whether this benefit was long lasting and also evident after BNT162b2 booster vaccination. Reactogenicity was classified into three groups: no or minor injection site symptoms, moderate (not further classified) and severe adverse reactions (defined as any symptom(s) resulting in sick leave). We initially compared 76 non-immunocompromised individuals who reported either no or minor injection site symptoms or severe adverse reactions after second vaccination. In total, 65 of them took part in another blood sampling and 47 were evaluated after booster vaccination. 26 weeks after second vaccination, men who reported severe adverse reactions after second vaccination had 1.7-fold higher SARS-CoV-2 RBD IgG titers (*p* = 0.025) and a 2.5-fold better neutralization capacity (*p* = 0.006) than men with no or only minor injection site symptoms. Again, no association was found in women. Reactogenicity of BNT162b2 booster vaccination was different from second vaccination according to our classification and was no longer associated with SARS-CoV-2 RBD IgG titers or wild-type neutralization capacity. To conclude, after BNT162b2 basic vaccination, the association between reactogenicity and humoral immune response in men persisted over time but was no longer detectable after BNT162b2 booster vaccination.

## 1. Introduction

In December 2020, the first COVID-19 vaccine BNT162b2 received emergency use authorization in the United States and Europe [1,2]. This was the beginning of a mass vaccination campaign unique in history and through a new era of vaccine research. One subject that was discussed controversially in the past was the question whether adverse reactions after vaccination are predictive signs of a desirable immune response [3]. We have recently conducted a study on the topic and found a highly significant association between severe adverse reactions defined as any symptoms resulting in sick leave after second BNT162b vaccination and anti-receptor binding domain (RBD) IgG titers and Wuhan pseudovirus neutralization capacity, yet only in men [4]. Meanwhile, there are numerous studies that mostly support and occasionally refuse an association between reactogenicity and immunogenicity of COVID-19 basic vaccinations [5,6,7,8,9,10,11,12,13,14,15].

There was an opportunity to continue research on this issue with the necessity of a booster vaccination that arose because of an increase in breakthrough infections and hospitalizations in vaccinated individuals with the enhanced transmission of the delta variant starting in spring 2021 [16,17].

In the present follow-up study, we therefore wanted to describe whether the previously found association between severe adverse reactions and humoral immune response in men is persistent and can again be reproduced after BNT162b2 booster vaccination.

## 2. Materials and Methods

### 2.1. Study Population

All 76 study participants included in the previous analysis were invited to participate in the follow-up study [4].

In brief, vaccinees were initially chosen according to the reactogenicity of the basic COVID-19 vaccinations. Reactogenicity information had been collected from 735 vaccinees from our University Medical Center vaccination clinic who had to classify adverse reactions after first and second vaccination separately *in no or minor* (defined as only local at injection site), *moderate* (not further classified) and *severe* (classified as any symptom(s) resulting in sick leave). Two groups were formed, the first consisting of vaccinees with *no or minor* adverse reactions after both vaccinations and the second with vaccinees who reported the most *severe adverse reactions* after first and second vaccination from the recruitment population. All participants of the latter group reported sick leave after second vaccination, whereas first vaccination could cause any of the three outcomes as sick leave was much rarer after first vaccination, especially in men. In total, 38 vaccinees were chosen from each group and were age- and sex-matched. No one was immunosuppressed.

From those who agreed to continue the study, another blood sample was taken to evaluate immunity over time. After booster vaccination, participants were invited to take part in a third blood sampling and to fill in a questionnaire that addressed reactogenicity of the booster vaccination. Only participants who received BNT162b2 as a booster vaccine were included in the analysis.

### 2.2. SARS-CoV-2 Binding Antibodies

Severe acute respiratory syndrome coronavirus type 2 (SARS-CoV-2) spike protein receptor-binding domain (Wuhan)-directed antibodies were detected using an in-house ELISA, as described previously [18]. For the determination of immunoglobulin G (IgG) antibody endpoint titers, serum samples were titrated in eight steps with twofold dilution, starting with a dilution of 1:200. Endpoint titers were calculated by using the parameters of a four-parameter logistic curve fit and a predetermined cutoff value.

### 2.3. SARS-CoV-2 Nucleocapsid Antibodies

To identify possible breakthrough infections that may have occurred since the initial vaccination, SARS-CoV-2 nucleocapsid-specific antibodies were detected using the Elecsys Anti-SARS-CoV-2 N assay (Roche Diagnostics GmbH, Penzberg, Germany). The assay has been shown to be highly sensitive and specific [19]. The measurements were performed on a Roche COBAS pro e 801 clinical chemistry analyzer according to the manufacturer’s recommendations.

### 2.4. Wild Type (Wuhan) Neutralization Test (NT)

SARS-CoV-2 neutralization tests were performed as described previously [19]. In brief, we used Vesicular Stomatitis Virus (VSV–Δ G*FLuc) [20] pseudotyped with SARS-CoV-2-Spike-ΔER (wildtype) and determined pseudoviral titers by limited dilution and fluorescence microscopy. For each sample, an inoculum of 25,000 fluorescence-forming units was neutralized in triplicate for 1 h with a 2-fold serum dilution series starting at 1/20. Luciferase activity was determined 20 h post-infection of HEK293T-ACE2 + -cells using BrightGlo (Promega Corp, Madison, WI, USA). IC_50_ values (50% maximal inhibitory concentration) were calculated using the algorithm: ‘log (inhibitor) vs. normalized response’ in GraphPad Prism 8 software (GraphPad Software, San Diego, CA, USA). Values above IC_50_ ≥ 20 were defined as positive.

### 2.5. Statistical Analysis

Statistical analysis was performed using Stata/BE 17.0. For paired values, Wilcoxon signed rank test was performed. For unpaired values, Mann–Whitney-U test was performed. For examination of paired ordered categorical data, Fleiss–Everitt-ordered categories chi2 test was used. *p* < 0.05 was considered significant. Data are presented as median (with interquartile range). Figures were constructed using GraphPad Prism version 9.2.0, GraphPad Software, San Diego, California USA.

The figures in the results section use stars to flag levels of significance. If a *p*-value is less than 0.05, it is flagged with one star (*). If a *p*-value is less than 0.01, it is flagged with 2 stars (**). If a *p*-value is less than 0.001, it is flagged with three stars (***).

### 2.6. Ethical Issues

The study was conducted according to the guidelines of the Declaration of Helsinki and was approved by the local Ethics Committee of the University of Regensburg (reference number: 21-2334_2-101). The study was registered under DRKS00026982. Informed consent was obtained from all subjects involved in the study.

## 3. Results

### 3.1. Participants

Seventy-six participants were included in the analysis of the previous study on immunogenicity and reactogenicity of BNT162b2 basic vaccination. The recruitment strategy is described in the methods section. In total, 65 of them took part in a further blood sampling at median 26 weeks (range 24.7–28.9) after second vaccination. Then, 47 took part in a third blood sampling at median four weeks (range 3.6–5.6) after booster vaccination with BNT162b2. There was no difference among the three groups in relation to age, sex, any chronic disease, smoking and body mass index (data not shown). No participant reported a history of SARS-CoV-2 infection, yet three vaccinees were nucleocapsid positive in first and second and two in booster analysis. Seven vaccinees were simultaneously boostered and vaccinated against seasonal influenza (Table 1).

### 3.2. Waning Immunity over Time and Immunogenicity of BNT162b2 Booster Vaccination

**Figure 1:**
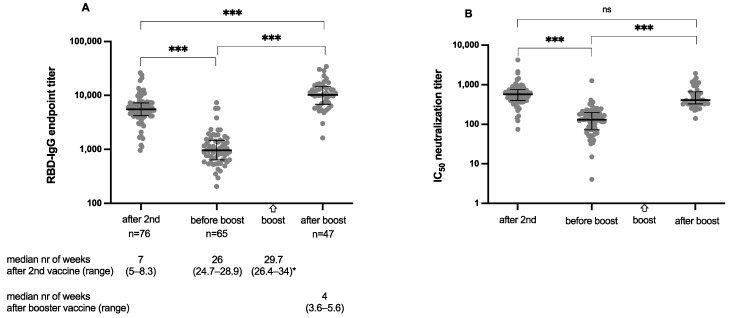
**Waning immunity over time and effect of booster vaccination:** 26 weeks after BNT162b2 basic immunization, receptor-binding-domain (RBD) IgG titers and neutralization capacity significantly decreased. By booster immunization, a significant increase in both parameters could be induced. Shown are individual IgG endpoint titers and median with interquartile range as measured by wild-type (WT) RBD (**A**) and neutralization capacity (**B**) given as half maximal inhibitory concentration (IC_50_) as measured by WT pseudovirus assay. For statistical analysis, Wilcoxon tests for paired values were performed. 2 missing values. *** *p* < 0.001; ns = not significant.

At a median interval of 26 weeks after second vaccination, SARS-CoV-2 RBD IgG titers decreased significantly from initially 5528 (range 958–26,285, interquartile range (IQR) 2993, n = 76) [4] to 961 (206–7370, 815, n = 65). By BNT162b2 booster vaccination, a 10.7 increase in SARS-CoV-2 IgG titers could be observed (median 10,284, 1629–34,394, 8157, n = 47) (Figure 1A). Similarly, neutralization capacity decreased from initially 577 (74–4231, 355) [4] to 132 (4058–1257, 116) and increased factor 3.1 after booster vaccination (median 412, 141–1930, 334) (Figure 1B). The booster vaccination led to significantly higher SARS-CoV-2 IgG titers compared to titers after second vaccination (*p* < 0.001); in contrast, neutralization capacity after booster vaccination was not different from second vaccination (*p* = 0.42).

### 3.3. Reactogenicity of Vaccinations

**Figure 2:**
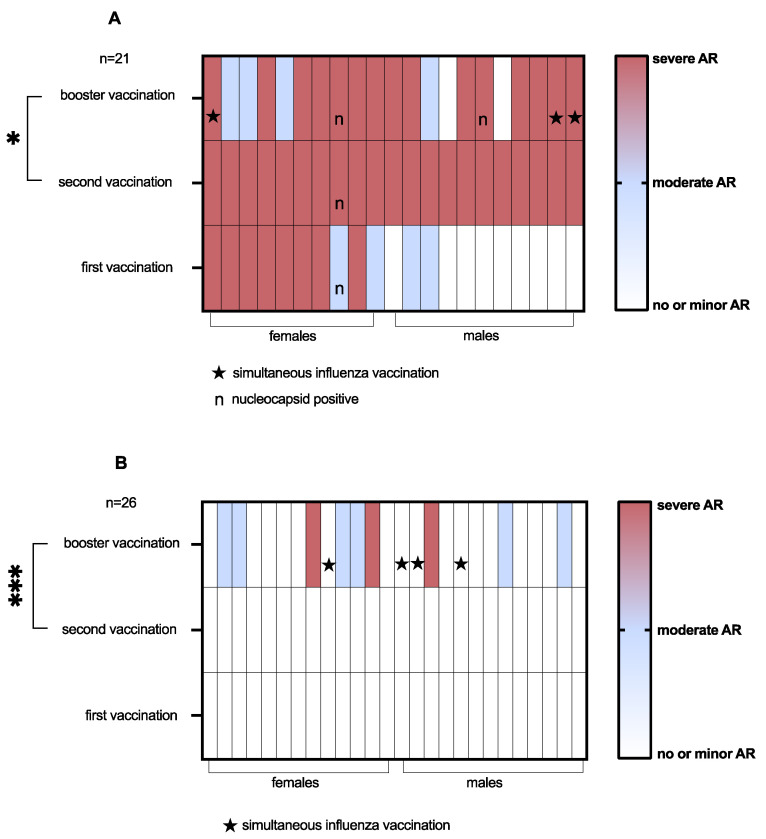
**Heat map of reactogenicity profiles.** After second vaccination, two groups were formed to compare vaccinees with most severe adverse reactions (**A**) with those who had no or only minor injection site symptoms (**B**). Shown are individual reactogenicity profiles after first, second and booster vaccination with BNT162b2. Subjects who received booster and influenza vaccination simultaneously are marked with asterisks, subjects with positive nucleocapsid measurements are marked with “n”. For statistical analysis, Fleiss–Everitt ordered categories chi2 test was used. AR = adverse reactions. * *p* < 0.05; *** *p* < 0.001.

Among vaccinees with most severe adverse reactions after basic vaccination (n = 21), booster vaccination caused again severe adverse reactions (sick leave) in 15 (71.4%) and moderate adverse reactions in 4 (19.0%) vaccinees. Two males (9.5%) had no or only minor injection site symptoms (Figure 2A). Change in reactogenicity category between second and booster vaccinations was significant (*p* = 0.02). All three vaccinees with simultaneous influenza vaccination had severe adverse reactions. One participant was nucleocapsid positive at all three timepoints, one only in blood sampling after booster vaccination.

Among vaccinees who reported no or only minor adverse reactions after both first and second vaccination (n = 26), three (11.5%) developed severe and six (23.1%) moderate adverse reactions after booster vaccination. The remaining 17 (65.4%) again reported no or only minor injection site symptoms; among them all 4 vaccinees of the group who were simultaneously vaccinated against seasonal influenza (Figure 2B). Change in reactogenicity category between second and booster vaccination was significant (*p* = 0.0047). No one was nucleocapsid positive at any timepoint.

**Figure 3:**
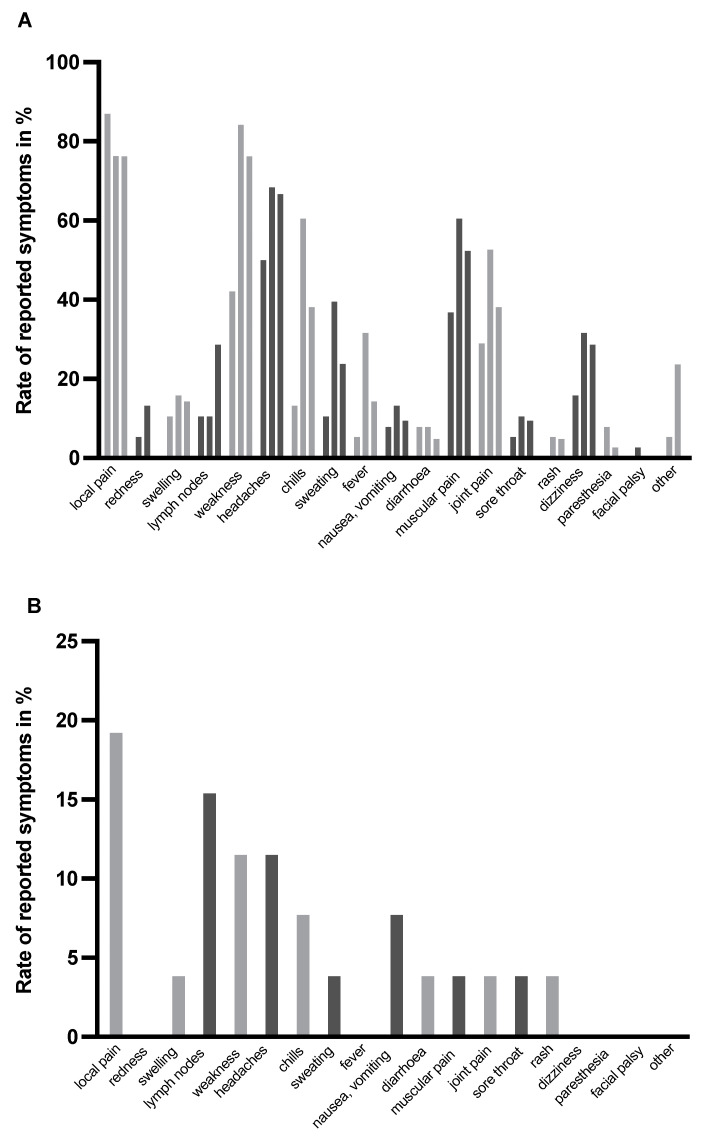
**Rate of adverse reactions after BNT162b2 basic and booster vaccination.** Panel (**A**) presents symptoms in vaccinees with most severe adverse reactions after basic vaccination (first and second bar, n = 38, respectively) and booster vaccination (third bar, n = 21); the single bar in Panel (**B**) presents symptoms in vaccinees after booster vaccination (n = 26) who had no or only minor injection site symptoms after first and second vaccination. Grey and black bars were chosen to differentiate the single symptoms and to make the figure more clearly arranged.

Figure 3 shows in detail what symptoms were reported by vaccinees after first, second and third vaccinations, according to initial classification. In vaccinees who suffered from severe adverse reactions after first and/or second vaccinations (Figure 3A), the three most frequent reported symptoms after each vaccination were the same: local pain (87.0/76.3/76.2%), weakness (42.1/84.2/76.2%) and headaches (50.0/68.4/66.7%). In vaccinees who had no or only minor injection site symptoms after first and second vaccinations, local pain (19.2%) and affection of lymph nodes (15.4%) were most frequently reported after booster vaccination, followed by weakness and headaches (11.5%, respectively) (Figure 3B).

### 3.4. Association of Immunogenicity and Reactogenicity

**Figure 4:**
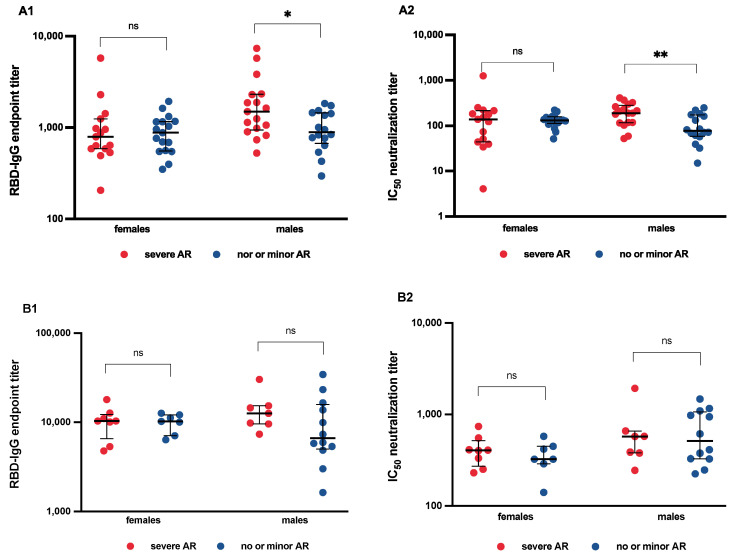
**Association of immunogenicity and reactogenicity.** Shown are antibody titers and neutralization capacity at median 26 weeks after second vaccination in 65 vaccinees of the initial reactogenicity groups (**A1**,**A2**) and at median 4 weeks after booster vaccination in 34 vaccinees with either severe adverse reactions or no or minor injection site symptoms after booster vaccination (**B1**,**B2**). Presented are individual IgG endpoint titers and median with interquartile range as measured by wild-type (WT) receptor-binding domain (**A**) and neutralization capacity (**B**) given as half maximal inhibitory concentration (IC_50_) as measured by WT pseudovirus assay. For statistical analysis, Mann–Whitney-U-Test was performed. AR = adverse reactions; ns = not significant. * *p* < 0.05; ** *p* < 0.01.

In our previous study, we found a significant association between reactogenicity and immunogenicity in men. Moreover, 27 weeks after second vaccination, this association was still detectable in men with severe adverse reactions, showing 1.7-fold higher SARS-CoV-2 antibody titers (median 1495 versus 895, IQR 1315 versus 737, *p* = 0.025) and 2.5-fold better neutralization capacity (median 189 versus 77, IQR 144 versus 110, *p* = 0.006). Again, adverse reactions did not influence antibody outcomes in females (Figure 4(A1,A2)).

For analysis of booster vaccination, out of 47 participants, we excluded three vaccinees who were simultaneously vaccinated against influenza and reported severe adverse reactions after vaccination. When analyzing the original reactogenicity groups, males with severe adverse reactions after booster vaccination no longer had higher SARS-CoV-2 RBD IgG titers or better neutralization capacity compared to men with no adverse reactions or only minor injection site symptoms. Again, no difference was found in females (data not shown).

We assumed that the most recent vaccination had the greatest impact on humoral immune response. Therefore, participants were newly grouped according to reported adverse reactions after booster vaccination. According to our initial strategy, to detect an influence of reactogenicity on immunogenicity, we only compared vaccinees that complained of severe adverse reactions resulting in sick leave with those who had no or only minor injection site symptoms after booster vaccination. Vaccinees with moderate adverse reactions after booster vaccination were therefore excluded. We finally included 15 vaccinees (8 females) with severe adverse reactions and 19 vaccinees (7 females) with no or minor adverse reactions. No significant association between reactogenicity and SARS-CoV-2 antibody titers and neutralization capacity could be found, either for all participants or for any sex (Figure 4(B1,B2)).

## 4. Discussion

The immunological benefit of higher SARS-CoV-2 antibody titers and neutralization capacity in men with severe adverse reactions (defined as any symptom(s) resulting in sick leave) after second vaccination with BNT162b2 [4] remained evident 26 weeks after second vaccination. At this timepoint, men with severe adverse reactions had 1.7-fold higher SARS-CoV-2 antibody titers and 2.5-fold better neutralization capacity compared to men with no or minor injection site symptoms.

Sex differences in immune response are well described, not only to vaccines but also to other foreign and self-antigens [21]. However, there are mostly females that report more frequent and severe local and systemic reactions to viral and bacterial vaccines and often have higher antibody responses [3,21]. We suppose that our approach of linking adverse reactions and sick leave may have been pivotal for our results, as adverse reactions are subjective and sick leave is possibly a more objective criterion.

After BNT162b2 booster vaccination, an association between adverse reactions and antibody results was no longer detectable in our study. Comparable data on the effects of the COVID-19 booster vaccination on this relationship could not be found. As we had a considerable number of “lost-to-follow-up” participants, it would have been difficult to detect small differences. Furthermore, after booster vaccination, reactogenicity classification changed for several individuals. Most interestingly, nine participants (six females) who had no or only minor injection site symptoms after first and second vaccinations now reported moderate or severe adverse reactions according to our classification. Vaccine reactogenicity is influenced by multiple factors; although individual factors did not change, there might have been a role for administration factors, psychological/physical stressors or circadian cycles [3].

Real-world data on reactogenicity of homologous BNT162b2 booster vaccination are inconsistent. A recent study described vaccine reactogenicity by using data from electronic health records. An increase in early postvaccination adverse events after the third dose compared with earlier doses was described; the symptoms were of low concern (i.e., fatigue, lymphadenopathy, nausea, and diarrhea) [22]. In a smartphone-based safety surveillance system in the United States, local and systemic adverse reactions in BNT162b2 vaccinees were less frequently reported following booster (64.3% and 58.4%, respectively) than following dose 2 (68.1% and 66.7%, respectively), (*p* < 0.001) [23]. In the United Kingdom ZOE COVID Study, the proportion of homologous BNT162b2 participants with systemic side-effects after the booster was slightly lower than after the second dose (13.2%, 95% CI 13.0–13·3, for the third dose vs. 19.2%, 19.0–19.4, for the second dose) [24].

The most frequently mentioned adverse reactions after BNT162b2 booster vaccination were pain or tenderness, malaise or fatigue, myalgia and headaches [24,25,26]. Similarly, pain, weakness and headaches were most often reported in our study; additionally, affection of local lymph nodes was an important symptom after booster vaccination in both groups.

SARS-CoV-2 antibody titers and neutralization capacity waned over time. By booster vaccination, a significant increase compared to pre-booster in SARS-CoV-2 antibody titers (10.7-fold) and neutralization capacity (3.1-fold) against wild-type virus could be achieved. Another study found an increase in wild-type neutralization capacity of more than 5-fold in 18-to-55 year-olds and more than 7-fold in 65-to-85 year-olds compared to one months after dose 2 [27]. A trial comparing homologous and heterologous booster vaccination described an increase in binding antibodies against the spike (S) protein with proline modification (S-2P) 14 days after homologous BNT162b2 booster of 15-fold, and an increase in neutralizing antibodies in pseudovirus D614G assay of 20-fold [25]. We suggest that the timing of blood sampling and assay application played an important role in different outcomes.

## 5. Conclusions

The previously found association between reactogenicity and antibody responses in men after basic vaccination with BNT162b2 remained evident over time. After BNT162b2 booster vaccination, reactogenicity in individuals changed and did no longer influence antibody results in vaccinees.

## Figures and Tables

**Table 1:** Baseline characteristics of study participants.

	After Second Vaccination	Before Booster Vaccination	After Booster Vaccination
Total number of participants	76	65	47
Age—years (median, range)	43 (23–64)	44 (23–64)	44 (23–64)
Sex—n (%) females	36 (47.4)	32 (49.2)	23 (48.9)
Any chronic disease—n (%)	19 (25.0)	18 (27.7)	15 (31.9)
Smoking—n (%)	5 (6.6)	5 (7.7)	5 (10.6)
Body-mass index (median, range)	24.4 (17.6–35.9)	24.6 (17.6–35.9)	24.5 (17.6–32.1)
Nucleocapsid positive—n (%)	3 (3.9)	3 (4.6)	2 (4.3)
Simultaneous influenza vaccination		7 (14.9)	

## Data Availability

The data sets used and/or analyzed during the present study are available from the first author on reasonable request.
